# An index for discrimination of mangroves from non-mangroves using LANDSAT 8 OLI imagery

**DOI:** 10.1016/j.mex.2018.09.011

**Published:** 2018-09-28

**Authors:** Kaushik Gupta, Anirban Mukhopadhyay, Sandip Giri, Abhra Chanda, Sayani Datta Majumdar, Sourav Samanta, Debasish Mitra, Rabindro N. Samal, Ajit K. Pattnaik, Sugata Hazra

**Affiliations:** a School of Oceanographic Studies, Jadavpur University, 188 Raja S. C. Mullick Road, Kolkata 700032, West Bengal, India; b Indian Institute of Remote sensing, Dehradun, 4, Kalidas Road, Uttarakhand, India; c Chilika Development Authority, Bhubaneswar, Odisha, India

**Keywords:** Combined Mangrove Recognition Index (CMRI), Mangrove discrimination, CMRI, NDVI, SR, SAVI, NDWI

## Abstract

Over the last few decades several vegetation indices were used to map Mangrove forest using satellite images. Difficulty still persists in discrimination of mangroves from non-mangrove vegetation, especially in areas where mangrove species are mixed with other vegetation types.

In the present study we have attempted to develop an improved index, which utilizes the information from the Normalized Difference Vegetation Index (NDVI) and the Normalized Difference Water Index (NDWI) of Bhitarkanika mangrove forest of Odisha, India. These indices are negatively correlated (r = –0.988; p < 0.01). Further, the NDWI values were subtracted from the NDVI values at the pixel level. As the outputs are negatively related, subtraction increases the upper and lower range of the overall output, also increasing the distinct values of two classes with near-similar spectral signatures. Same algorithm was applied on mangroves of Sundarbans (r = −0.987) and Andaman (r = −0.989).

A comparison between four established indices [NDVI, NDWI, Soil Adjusted Vegetation Index (SAVI), Simple Ratio (SR)] and the newly developed index namely Combined Mangrove Recognition Index (CMRI) were performed. Accuracy assessment using Kappa statistics, revealing that CMRI produces better accuracy (73.43%) compared to other indices, followed by NDVI (56.29%) and SR (48.79%).

## Graphical abstract

**Figure:**
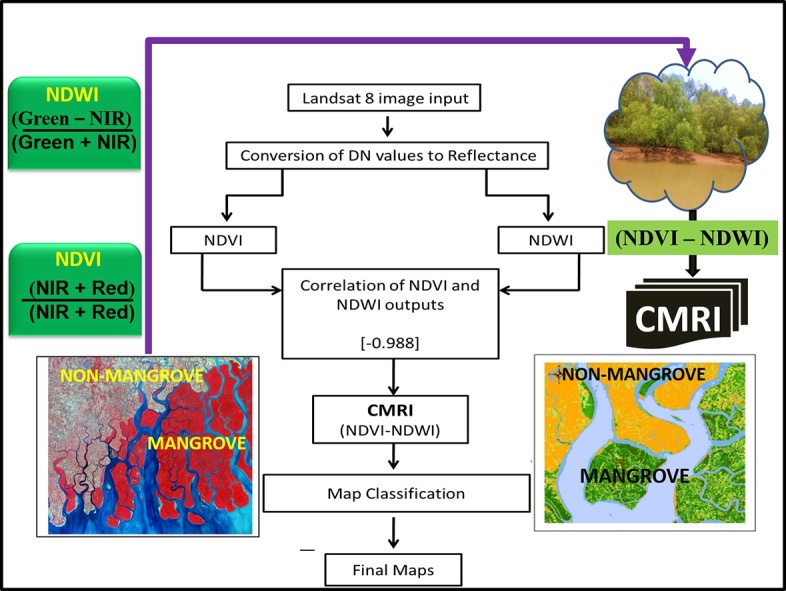

**Specifications Table**

**Table:** 

Subject area	*Environmental Science*
More specific subject area	*Remote Sensing*
Method name	Combined Mangrove Recognition Index (CMRI)

## Method details

### Rationale

Mangrove forests are one of the most bio-diverse ecosystems along tropical seacoasts and estuaries consisting of salt-tolerant plants with aerial breathing roots that work as sediment entrapments and provide a microenvironment to many marine species [1,2]. Mangrove helps to regulate coastal flooding and erosion, as well as protect inland agricultural fields, livestock and homesteads and other near shore communities from natural hazards like cyclones and hurricanes [3,4]. It supports a diverse group of flora and fauna in both the terrestrial and aquatic compartments of mangrove ecosystem [4]. Mangroves play an important role in coordinating a source and sink system for many biochemical substances, such as atmospheric carbon di-oxide, their transformation, accumulation and remediation [[5], [6], [7]]. In addition to these, mangroves also directly contribute to the economy and livelihood of coastal communities by providing honey, fuel, traditional medicine and also acting as potential ground for aquaculture and fisheries [3,4].

It has been long recognized that most of the areas rich in mangrove diversity are predominantly inaccessible or logistically difficult to study on field and at the same time substantially time taking. Hence there was a demand of a better, cost effective and less time consuming method of studying mangrove ecosystems [8,9]. Over the last few decades remote sensing technique has been applied as an effective tool for regular monitoring of mangrove forest and providing scope of studying areas that are truly inaccessible and remote ([[10], [11], [12]]; [52], [[13], [14], [15]]). Several attempts of mangrove classification and mapping have been made in India [[16], [17], [18], [19], [20], [21]]. Though remote sensing data does not completely replace the ground truth verification, yet the use of remote sensing data is advantageous in obtaining quick synoptic coverage having high temporal resolution [17,[22], [23], [24]] enabling change detection studies much easier than field based estimates.

The aim of this study was to employ spectral signatures and morphological characteristics of mangroves to generate an improved index for separating mangrove vegetation from non-mangrove vegetation classes and to compare the performance of the index with other established vegetation discriminating indices [(e.g. Normalized Difference Vegetation Index (NDVI), Normalized Difference Water Index (NDWI), Soil Adjusted Vegetation Index (SAVI), Simple Ratio (SR)] using LandSat 8 OLI imagery. The new index developed in this study namely ‘Combined Mangrove Recognition Index (CMRI)’ incorporates outputs from NDVI and NDWI indices in order to assess exclusively the mangrove vegetation using information like greenness and water content (succulence). The study has been carried out in three major mangrove forests of India namely Sundarban mangroves, Bhitarkanika Mangroves and the mangroves of Andaman.

Mangrove Recognition Index (MRI) [25] was developed with a similar aim of separating mangrove vegetation from non-mangrove vegetation in Beilunhekou National Nature Reserve Area of China. The index uses greenness and wetness index values collected from satellite data considering both high and low tide conditions, as the author explains there are changes in spectral signature during each event. However, the tidal conditions, salinity and surrounding vegetation diversity vary a lot all over the world. Under these circumstances an index independent of any particular condition will be more appropriate. In our paper mangroves have been differentiated using leaf water content and overall health condition (greenness) and the methods have been validated in three different geo-physical conditions such as Sundarban, located in the Ganga-Brahmaputra-Meghna (GBM) delta region located in the coastal areas, Bhitarkanika a land confined mangrove forest and Andaman, an island region with different tidal amplitude and salinity. The proposed index is useful for discriminating mangroves in different locations all over the world.

### Methodology

#### Description of study area

**Fig. 1:**
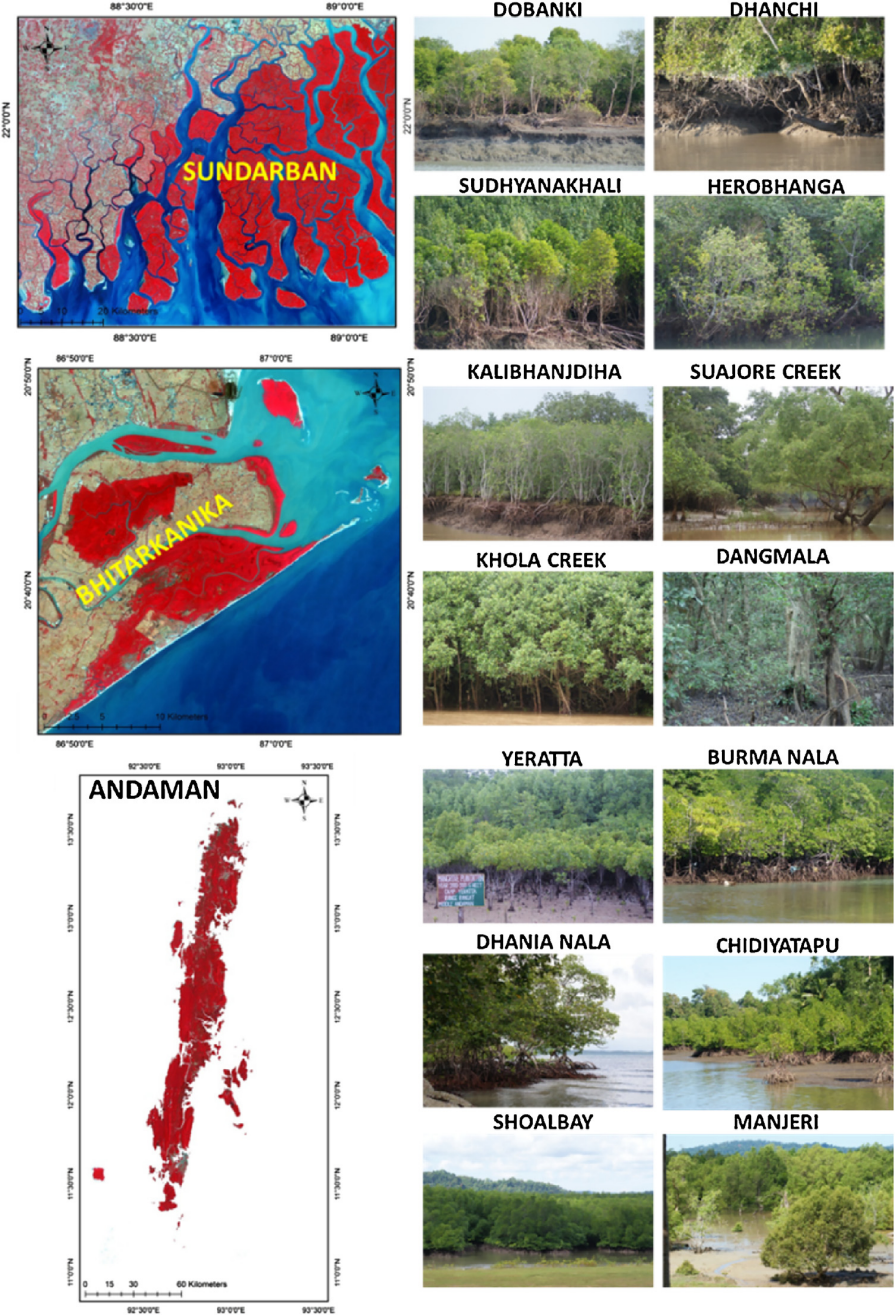
Study area map showing the Sundarban mangroves (Abundant species are *Avicennia* sp., *Bruguiera* sp., *Ceriops* sp., *Excoecaria* sp., *Sonneratia* sp.), Bhitarkanika mangroves (Abundant species are *Avicennia* sp., *Heritiera* sp., *Excoecaria* sp.) and Andaman mangroves (Abundant species are *Avicennia* sp., *Rhizophora* sp., *Lumnitzera* sp.).

The locations selected for this study namely, Indian Sundarbans, West Bengal; Bhitarkanika National Park, Odisha and Andaman Islands (Fig.1) represent three significantly different saline habitats. Andaman Islands represent a typical tropical island system devoid of much sediments and freshwater flow, while Sundarbans and Bhitarkanika represent estuarine habitat of contrasting salinities. While macrotidal Indian Sundarbans with lesser freshwater supply appears as a highly saline habitat [26], Bhitarkanika estuary offers a more freshwater dominated habitat for the mangroves [27].

The Indian Sundarbans mangrove forest covering an area around 2100 km^2^ is one of the most diverse ecosystems in terms of flora and fauna also representing one of the World’s largest mangrove forests ([50]; [[28], [29], [30]]). Sundarban is highly sensitive to the climate variability and increasing population density [31]. Coastal erosion and inundation [32] are the dominant threats to the Sundarbans due to loss of sediment supply and sea level rise in the Bay of Bengal [[33], [34], [35]]. Huge siltation in the different distributaries of river Ganges has resulted in the decrease of freshwater flow in the Sundarbans and increase in salinity in last few decades [36]. Thus, high salinity tolerant mangrove species like *Avicennia alba* and *Avicennia officinalis* are gradually replacing the freshwater loving species like *Heritiera* sp. [37,38]. Sundarbans is dominated by an assemblage of species like *Avicennia* sp., *Excoecaria* sp. and *Sonneratia* sp.

Bhitarkanika National park, Odisha is another mangrove habitat of around 130 km^2^ area situated in the downstream of Mahanadi basin [39]. The assemblage of species found in Bhitarkanika is more or less consistent throughout the National park. The river banks and intertidal areas are mostly dominated by species like *Avicennia* sp. and *Excoecaria* sp., followed by a thick monocrop patch of *Heritiera* sp. in the inland regions [1,40].

Andaman Islands exhibit a typical island habitat for mangroves of Indo-Pacific affinity. The mangroves occur along tidal inlets and few mudflats, often along sandy or rocky coasts sheltered by fringing coral reefs. Andaman Islands have around 430 km^2^ of mangrove cover. Andaman Islands are dominated by *Rhizophora* sp., followed by *Bruguiera* sp. and *Sonneratia* sp. [41] with occasional patches of only mangrove palm (*Nypa fruticans*) along inland creeks.

These three regions were selected for the study as they differ in species diversity and types of species assemblage. The geomorphological set up of these three locations are quite different especially from the perspective of salinity regime and freshwater flow. Sundarban mangroves experience very high salinity due to scarcity of freshwater flow from the upstream. Bhitarkanika on the contrary, receives substantial amount of freshwater from the distrbutaries of Mahanadi River and hence experiences low to moderate salinity. Andaman on the other hand stands as an example of mangroves growing in island periphery having rocky substratum and experiences quite high salinity. The tidal nature is also quite different in these three regions. Sundarban, Bhitarkanika and Andaman witnesses a meso-macro tidal, meso tidal and micro tidal environment respectively. From the perspective of species composition, Bhitarkhanika National Park comprises several monocrop assemblages of true mangrove species with negligible mixing of mangrove and non-mangrove canopies. In contrast to Bhitarkhanika, numerous mangrove-dominated areas in the buffer zone of the Sundarban exhibited a high level of mixing of mangroves and non-mangroves in the peripheral regions creating a mixed canopy. In case of Andaman Islands, particularly in the western coast of North and Middle Andaman, classification of mangroves is a challenge as the elevation throughout the coast creates a shade effect on the underlying vegetation types, thus imparting a spectral signature similar to mangrove canopies. All these physical settings are unique in their respective regions and most essentially covers all the types of Indian mangroves as well as the geophysical set up of the global mangrove cover.

#### Collection of Ground Control Points (GCP)

A total of 285 GCPs were collected from the three study sites: Andaman Islands (127), Bhitarkanika (67) and Indian Sundarbans (91). The points were collected majorly from peripheral patches of mangroves to provide more ground information for separating mangroves from non-mangrove vegetation. Some points were deliberately taken from sites with a dense mangrove dominated areas. GCPs in Bhitarkanika were collected from areas like Dangmala, Kalibhanjhdiha and Suajore Creek. In Sundarbans, Jharkhali, Dhanchi, Chotorakhashkhali, Koikhali areas were visited for collection of ground information. GCP locations in Andaman were spread across Aerial Bay, Rangat, Kadamtala, Baratang, Shoal Bay, Chouldari, Wandoor, Manjery, Chidiyatapu and Bambooflat.

#### Data used

The study was conducted using Landsat 8 OLI multispectral imagery covering the areas of Andaman Is, Bhitarkanika and Indian Sundarbans. Three bands (Red Band, Green Band and Near Infra-Red Band) out of the eight bands (Coastal Aerosol, Blue, Green, Red, NIR, SWIR 1, SWIR 2 and Cirrus) were used for all classification and interpretation techniques. Table 1 shows the details of the satellite images used for the study.

**Table 1:** Description of Data used.

Sl. No	Location	Sensor	Date of Acquisition	Spatial Resolution	Path	Row
1.	Andaman Is (Image 1)	Landsat 8 OLI	28.03.2017	30 m	134	51
2.	Andaman Is (Image 2)	Landsat 8 OLI	28.03.2017	30 m	134	52
3.	Bhitarkanika	Landsat 8 OLI	01.01.2017	30 m	139	046
4.	Sundarbans	Landsat 8 OLI	03.01.2018	30 m	138	045

#### Pre-processing of data

Like other Landsat images, Landsat OLI also requires radiometric calibration. Gain bias correction, scattering effect and correction of the sun angle [42,43] are most important for vegetation analysis using LANDSAT imageries. In the present study Green, Red and NIR bands of the Landsat OLI have been atmospherically corrected. The bands were radiometrically calibrated to convert DN values into top of the atmosphere (TOA) spectral radiance. The factors are provided in the metadata file used for the correction. Subsequently, the bands were converted to TOA planetary reflectance. The DN values were converted into TOA reflectance without sun angle correction using the following formula ([51] Users Handbook, 2016) using the below mentioned formulae:

*ρλ^'^* = *M_ρ_Q_cal_* + *A_ρ_*

where ρλ' = TOA planetary reflectance, without correction for solar angle. Note that ρλ' does not contain a correction for the sun angle.

Mρ = Band-specific multiplicative rescaling factor from the metadata (REFLECTANCE_MULT_BAND_x, where x is the band number)

Aρ= Band-specific additive rescaling factor from the metadata (REFLECTANCE_ADD_BAND_x, where x is the band number)

Qcal = Quantized and calibrated standard product pixel values (DN)

Now the Sun Angle correction have been achieved using the following formula

$$\rho \lambda =\frac{\rho \lambda^{\prime }}{\cos\left(\theta_{SZ}\right)}=\frac{\rho \lambda^{\prime }}{\sin\left(\theta_{SE}\right)}$$

Where:

ρλ = TOA planetary reflectance θ_SE_ = Local sun elevation angle. The scene center sun elevation angle in degrees is provided in the metadata (SUN_ELEVATION). θ_SZ_ = Local solar zenith angle; θ_SZ_ = 90° − θ_SE_

#### Image classification

**Fig. 2:**
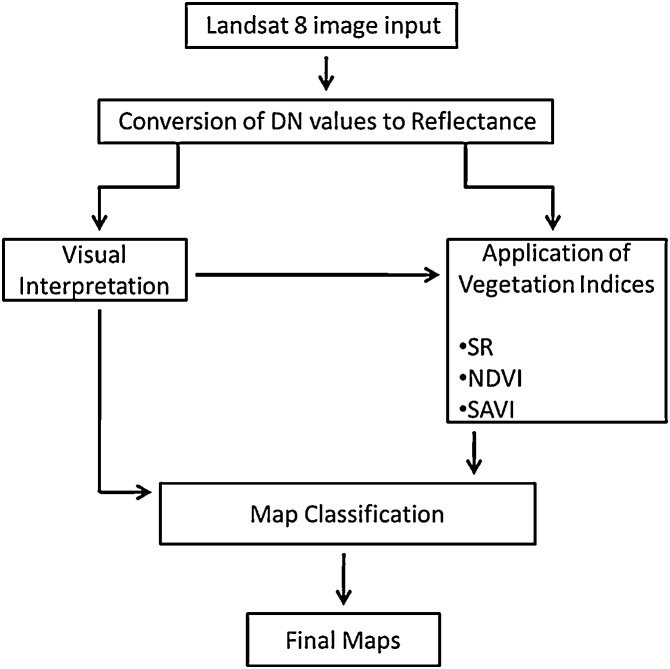
Primary process flow chart.

Conversion of DN values to radiance helps us acquire and understand the spectral properties of vegetation types better. Further analysis was carried out on this corrected datasets. The combination of three Landsat OLI bands and the indices were applied (Fig. 2) and tested on the Bhitarkanika image using available ground information. The classes were separated into four main groups including: (i) mangrove dominated class, (ii) non-mangrove vegetation class, (iii) non-vegetation class (including barren land, settlements, mudflats and beaches) and (iv) water dominated class.

#### Development of CMRI

Water classification index NDWI was applied on the images. It was observed that the product provides a coarse distinction in signatures of mangrove dominated areas, as mangroves exhibit a property of high water content in its leaves, which is to an extent exploited using this index. Whereas, NDVI uses the greenness of leaves and its absorption and reflection of Red and NIR band to extract information based on the plant chlorophyll content. In order to generate an index to distinguish mangroves from other vegetation types using both the above mentioned indices sensitive to mangroves, we found the relation between the two outputs obtained from the classification.

**Fig. 3:**
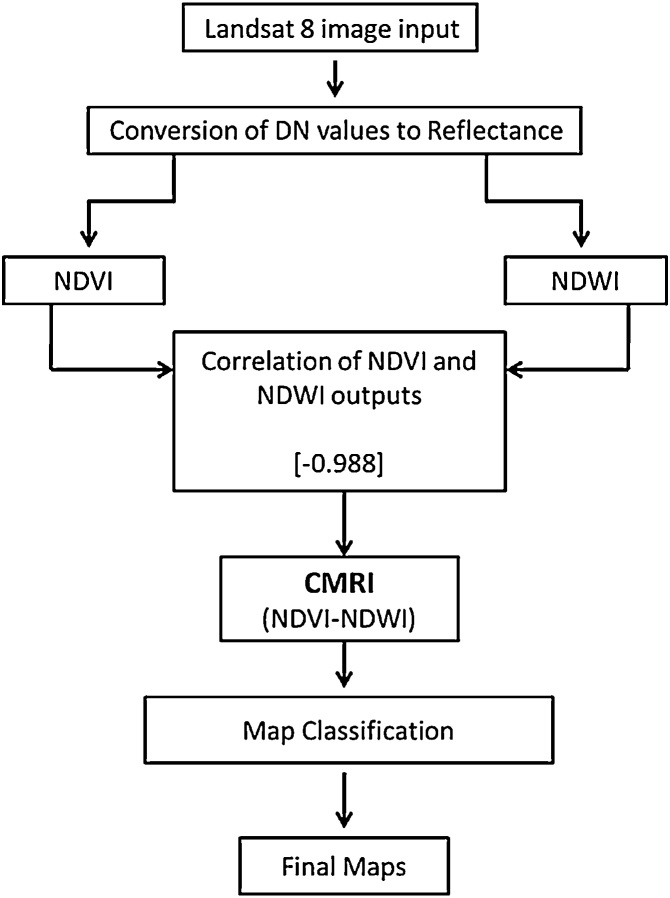
A flow chart diagram showing the generation of Combined Mangrove Recognition Index (CMRI).

A correlation of the NDVI and NDWI outputs for Bhitarkahnika was performed. The results showed that they were negatively correlated (r = −0.988), with strong inverse relationship. A simple algorithm was used, subtracting the NDWI values from the NVDI values at pixel level (Fig. 3). As the outputs are negatively related, subtraction was found to increase the upper and lower range of the overall output, eventually increasing the scope of distinction between two classes with near-similar spectral signatures. Later this algorithm was applied on Indian Sundarbans and Andaman Islands as well to test its robustness in a different type of mangrove habitats with a greater heterogeneity of vegetation types. Equally strong, negative correlation was found in both the places (r = −0.987 and r = −0.989 respectively), between the two indices of NDVI and NDWI. Details of the various pre-established indices and the new derived indices used in the study are given in Table 2.

**Table 2:** Description of Indices used.

Class	Formulas	Reference
Simple Ratio (SR)	(NIR/Red)	[44]
Normalized Difference Vegetation Index (NDVI)	$\frac{\left(\text{NIR}-\text{Red}\right)}{\left(\text{NIR}+\text{Red}\right)}$	[45]
Soil Adjusted Vegetation Index (SAVI)	$\frac{\left(\text{NIR}-\text{Red}\right)\left(1+\text{L}\right)}{\left(\text{NIR}+\text{Red+L}\right)}$	[44]
Normalized Difference Water Index (NDWI)	$\frac{\left(\text{Green}-\text{NIR}\right)}{\left(\text{Green}+\text{NIR}\right)}$	[46]
Combined Mangrove Recognition Index (CMRI)	(NDVI – NDWI)	Present study

#### Accuracy assessment

Further the 285 ground control points were plotted using ArcGIS 10.3. Item description according to the vegetation type observed on field was added to all the points, separating them into two categories of mangroves and non-mangroves. Information of the vegetation types from the outputs of the five performed indices were also classified into this two classes and distinct color was designated (red for mangroves and green for non-mangrove vegetation types). The accuracy of the indices was calculated using *Kappa* statistics, generating overall classification accuracy.

### Method validation

**Fig. 4:**
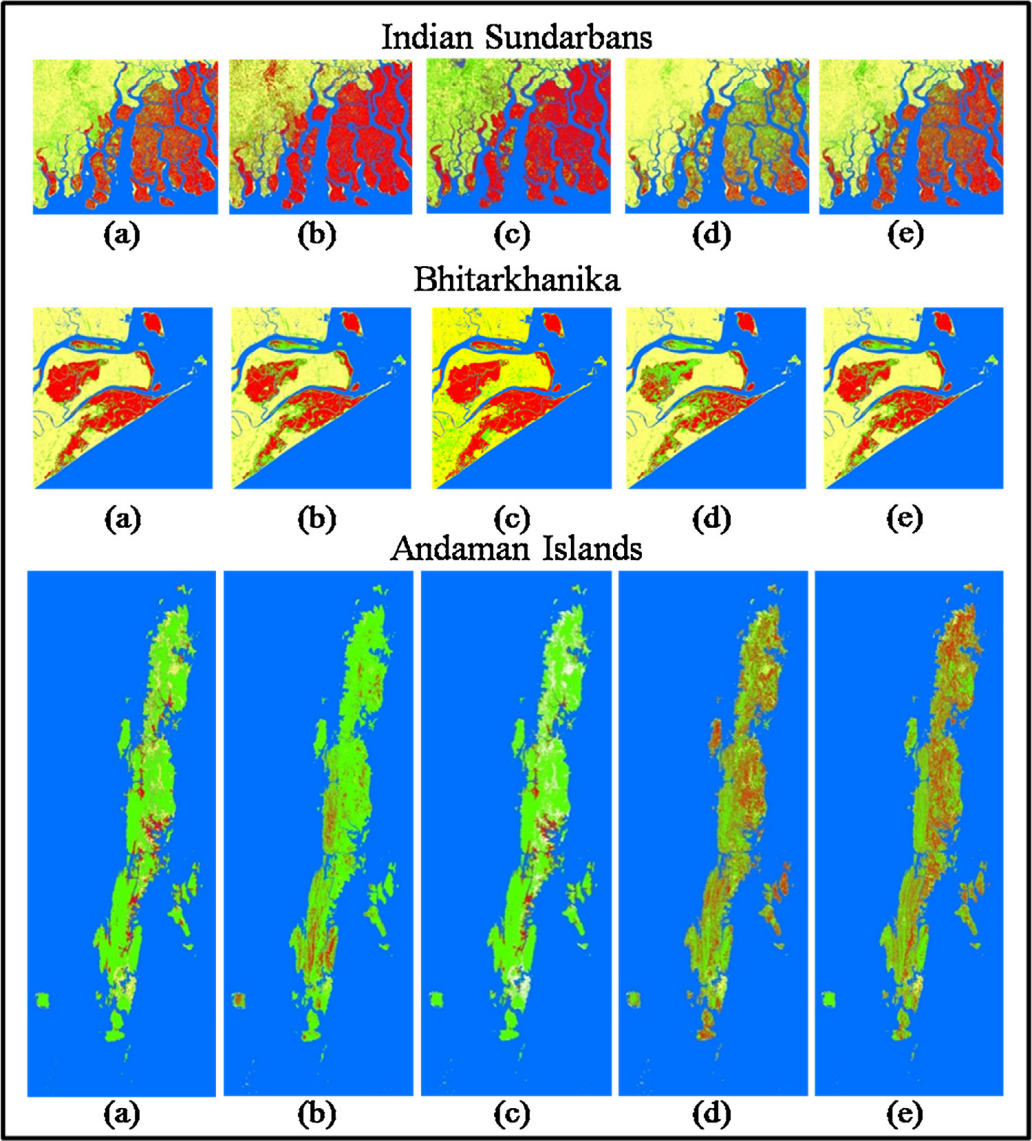
Classification outputs of (a) CMRI, (b) NDVI, (c) NDWI, (d) SAVI and (e) SR on Indian Sundarbans, Bhitarkanika and Andaman Islands. [Here the features are separated as Water (Blue), Land (Yellow), Non-mangrove Vegetation (Green) and Mangrove (Red)].

All five outputs generated from the indices SR, NDVI, NDWI, SAVI and CMRI using LandSat 8 OLI imagery were tested using available ground information in Bhitarkanika, Sundarbans and Andaman Island of India. Classification from the output images using the indices were categorized into four color designated classes: (i) water (Blue), (ii) land (Yellow), (iii) non-mangrove vegetation (Green) and (iv) mangroves (Red) (Fig. 4).

Mangroves have high water content in their leaves which enables them to thrive properly under high saline conditions. Mangroves exhibit substantial tolerance to a wide range of soil salinity [47]. Saline soil in comparison to non-saline soil offers a higher physiological challenge to the plants due to the highly negative water potential of soil pore water, making water acquisition a greater energy involved process [53]; [48]). In order to sustain in high salinity and high energy conditions, mangroves have evolved a number of adaptations like alterations of leaf size and angle, succulence or water storage in leaves, suberization of roots and biomass partitioning [53]. In mangroves it has been observed that high soil salinity increased leaf water content [48,49]. Succulence of leaves enable mangroves to impound large amounts of solutes to maintain turgidity at low water potential and without adversely increasing cell osmotic pressure [49]. We observed in this study that mangrove dominated areas exhibited an exclusive range of NDWI values in all the three study sites due to the comparatively higher water content in the mangrove leaves with respect to other vegetation types.

The classification outputs were further subjected to accuracy assessment using Kappa statistics. Table 3 shows that Combined Mangrove Recognition Index produces a higher overall classification accuracy compared to the other indices applied on Andaman Islands, Indian Sundarbans and Bhitarkanika (86.72%, 73.12% and 60.44% respectively) reserve forest. Average classification accuracy (Table 3) over all the three study areas shows CMRI having a better potential followed by NDVI and SR (73.43%, 56.29% and 48.79% respectively) for discrimination of mangroves from non-mangroves.

**Table 3:** Overall classification accuracy (OCA) using Kappa statistics and their averages over Sundarbans, Bhitarkanika and Andaman Is.

Indices	Sundarbans	Bhitarkanika	Andaman Is.	Avg. OCA in (%)
Simple Ratio	42.86	58.21	45.31	48.79
SAVI	27.47	26.87	47.66	34.00
NDVI	56.04	59.7	53.13	56.29
NDWI	53.85	16.42	64.84	45.04
CMRI	60.44	73.13	86.72	73.43

Discriminating mangroves with a high greenness values and high water content in their leaves, from non-mangrove vegetation have always been a problematic issue. Unless validated by meticulous ground level information in the mixing zone, remote sensing studies may lead to an overestimation of such forest cover. A comparatively accurate semi-automatic discrimination of mangrove classes from non-mangrove vegetation types in the Bhitarkanika, Sundarbans and Andaman Islands, India has been achieved in the present study by the development of Combined Mangrove Recognition Index (CMRI) using Landsat 8 OLI multispectral imagery. The index derives the information of the NDVI and NDWI products to furnish its own range of classes for a fine distinction of mangrove species from other vegetation types.

The western coast of the North and Middle Andaman Islands, has higher elevations which creates a shade on the underlying zones during satellite pass in the morning. This in effect creates an anomaly when applying a semi-automatic classification technique for discriminating mangroves from non-mangrove vegetation. However, CMRI demonstrated least sensitivity to this error.

The present index therefore provides us with a wide range of signature for mangroves, which can be used successfully to overcome such difficulty in estimation of mangrove cover. Additionally, the index can be applied in the future for the study of mangrove species diversity based on spectral properties of the canopy, with a better ground information for training the model, high resolution imagery and sub-pixel level classification. The indices generated in the study uses the Green, Red and NIR band from the Landsat 8 OLI mission. The range of wavelengths of these bands are 0.533–0.590, 0.64–0.67 and 0.85–0.87 (Green, Red and NIR respectively). This range of wavelength is available in most multispectral missions like Sentinel 2, SPOT MS, RESOURCESAT and other Landsat missions. Hence this index can be applied on satellite images from other sensors as well.
